# Self-Assembled Supramolecular Ribbon-Like Structures Complexed to Single Walled Carbon Nanotubes as Possible Anticancer Drug Delivery Systems

**DOI:** 10.3390/ijms20092064

**Published:** 2019-04-26

**Authors:** Anna Jagusiak, Katarzyna Chłopaś, Grzegorz Zemanek, Małgorzata Jemioła-Rzemińska, Barbara Piekarska, Barbara Stopa, Tomasz Pańczyk

**Affiliations:** 1 Chair of Medical Biochemistry, Faculty of Medicine, Jagiellonian University Medical College, Kopernika 7, 31-034 Krakow, Poland; katarzyna.chlopas@gmail.com (K.C.); grzegorz.zemanek@uj.edu.pl (G.Z.); barbara.piekarska@uj.edu.pl (B.P.); mbstopa@cyf-kr.edu.pl (B.S.); 2 Department of Plant Physiology and Biochemistry, Faculty of Biochemistry, Biophysics and Biotechnology, Jagiellonian University, Gronostajowa 7, 30-387 Krakow, Poland; malgorzata.jemiola-rzeminska@uj.edu.pl; 3 Malopolska Centre of Biotechnology, Jagiellonian University, Gronostajowa 7a, 30-387 Krakow, Poland; 4 Institute of Catalysis and Surface Chemistry, Polish Academy of Science, Niezapominajek 8, 30-239 Krakow, Poland; panczyk@vega.umcs.lublin.pl

**Keywords:** self-assembled ribbon-like structures (SRLS), Congo Red (CR), single walled carbon nanotubes (SWNT), doxorubicin (DOX)

## Abstract

Designing an effective targeted anticancer drug delivery method is still a big challenge, since chemotherapeutics often cause a variety of undesirable side effects affecting normal tissues. This work presents the research on a novel system consisting of single walled carbon nanotubes (SWNT), dispersed with Congo Red (CR), a compound that forms self-assembled ribbon-like structures (SRLS) and anticancer drug doxorubicin (DOX). SWNT provide a large surface for binding of planar aromatic compounds, including drugs, while CR supramolecular ribbon-like assemblies can be intercalated by drugs, like anthracycline rings containing DOX. The mechanism of interactions in SWNT–CR–DOX triple system was proposed based on electrophoretic, spectral, Dynamic Light Scattering and scanning electron microscopy analyzes. The profile of drug release from the investigated system was evaluated using dialysis and Differential Scanning Calorimetry. The results indicate that ribbon-like supramolecular structures of CR bind to SWNT surface forming SWNT–CR complexes which finally bind DOX. The high amount of nanotube-bound CR greatly increases the capacity of the carrier for the drug. The high capacity for drug binding and possible control of its release (through pH changes) in the analyzed system may result in prolonged and localized drug action. The proposed SWNT–CR–DOX triple system meets the basic criteria that justifies its further research as a potential drug carrier.

## 1. Introduction

Chemotherapy, widely used in cancer treatment, is efficient in many cases but carries a high risk because of a poor selectivity of chemotherapeutics. Due to side effects of these drugs on healthy tissues, solutions that could reduce their toxicity are constantly searched for not only among dose-lowering strategies but also among targeted drug delivery technologies which allow for maintaining therapeutic drug doses. Appropriate carriers serve to overcome the difficulties limiting the use of some drugs, including the lack of selectivity, low solubility at physiological conditions, limited penetration through cell membranes [1], and most of all, inability to surmount multidrug resistance (MDR) of cancer cells [2]. Comparisons of the effects of drug administration with and without the carrier demonstrated many advantages of drug carrier systems, which include, among others, selective biodistribution, high capacity and controlled release. Drug carrier systems have been successfully used for transport of therapeutically active molecules, like drugs [3,4,5,6], nucleic acids [7,8], peptides [9], proteins [10] and others. For some time, carbon nanotubes (CNT) have been described as a candidate carrier for chemotherapeutics [11,12,13].

Studies of carbon-based materials, in particular carbon nanotubes, are currently in progress to test their usefulness as potential drug carriers. Nanotubes are built of folded graphene layers. Very strong carbon-carbon bonds provide them with an exceptional property, which is a high strength-to-weight ratio [14,15,16,17]. Hybridization of sp^2^ type facilitates easy binding of different molecules on their surface. This is an advantage of nanotubes over other drug carriers (such as liposomes, dendrimers or nanoparticles). CNT show a wide range of applications, and studies on their use are carried out in different fields including materials science and medicine. Their universal character results from physicochemical properties: light weight, high strength-to-weight ratio, ordered structure and high length-to-cross section ratio. The diameter of single-walled carbon nanotubes (SWNT) ranges from 0.4 to 3 nm and the length reaches several micrometers [18,19].

CNT are an interesting nanomaterial especially because of their small dimensions at large surface area—from about 1000 m^2^/g to a maximum of 2600 m^2^/g [20] and their mechanical properties (especially a high strength at a large elasticity) [21]. These properties can contribute to the prolongation of the circulation time of nanotubes used as drug carriers thus increasing bioavailability of their content. However, disadvantages of nanotubes include accumulation in tissues and difficulties with their removal from the body. For this reason, the search for compounds helpful for dispersion of nanotubes in aqueous solutions and facilitating their excretion from the body is justified.

Supramolecular ribbon-like structures (SRLS) are another type of potential carrier system for chemotherapeutics [22]. One example of such compounds, Congo Red (CR), has been commonly known and used almost for a century as a histochemical dye used to detect amyloids, which after staining present as an apple-green birefringence under a polarization microscope.

The Congo Red molecule is characterized by symmetry, the presence of a central hydrophobic area and naphthalene rings substituted by amine and sulfonyl groups. The presence of azo bonds linking naphthalene rings with central benzidine is the basis for classification of Congo Red into the group of the so-called bisazo dyes. Benzidine aromatic rings of Congo Red participate in binding to non-polar nanotube surface. Doxorubicin, also possessing a planar aromatic structure, binds directly to CNT surface. The present study attempted to explain the mechanism of interaction between CR, DOX and SWNT.

**Figure 1:**
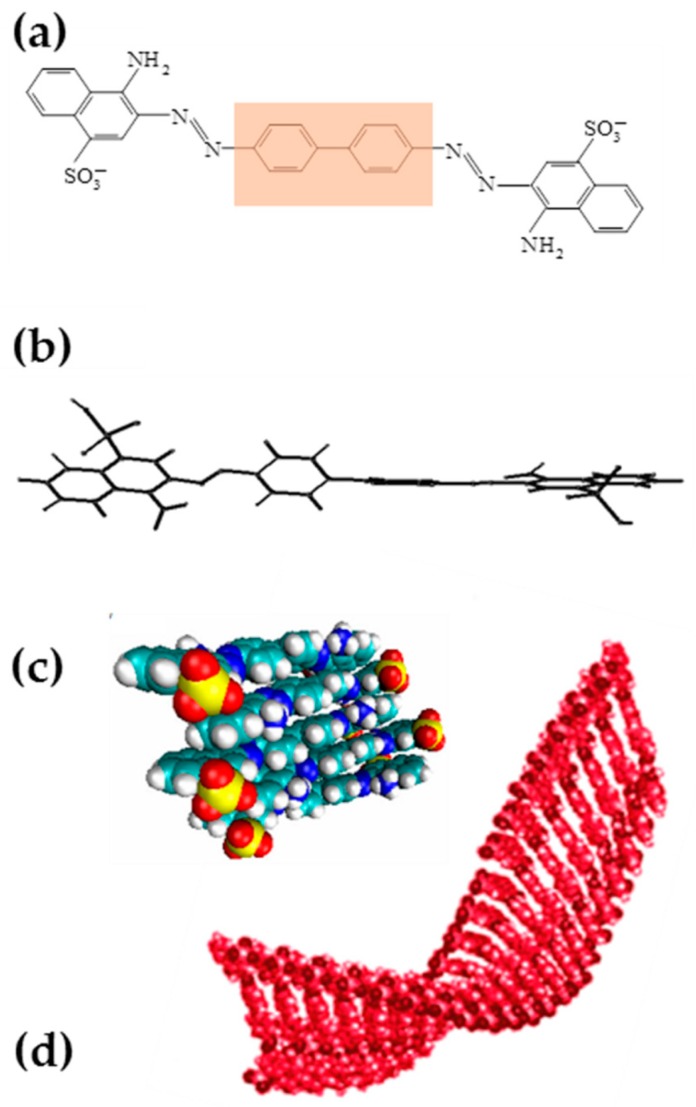
Congo Red (CR): (**a**) Chemical structure (the hydrophobic area is marked in beige), (**b**) conformation, (**c**) CR self-assemblies formed in water solutions by stacking of planar molecules, (**d**) high plasticity supramolecular ribbon-like system resulting from association of CR molecules.

In aqueous solutions Congo Red forms multimolecular aggregates called supramolecular systems, where molecules of the dye interact creating a ribbon-like structure. The presence of sulfonyl groups renders these structures polyanionic at neutral pH. The hydrophobic core of the elongated supramolecular structure is created by parallel-oriented interacting aromatic rings while sulfonyl groups are exposed outward towards aqueous environment. CR molecules interact face-to-face, engaging π electrons. In such a system, charge delocalization can occur, leading to possible formation of a dipole within CR supramolecular ribbon [22,23,24,25] (Figure 1).

CR type SRLSs bind to the surface of carbon nanotubes facilitating their efficient dispersion in aqueous solutions [26,27,28], which was presented in an earlier paper [29]. The mechanism of non-covalent interaction of CR-type compounds with SWNT surface was suggested to involve face-to-face interaction of CR molecules with nanotube surface. SRLS can also bind guest-like molecules possessing a flat ring non-polar region. The binding occurs via their intercalation into the ribbon structure. Earlier studies demonstrated that CR could bind e.g., Evans blue (which led to shortening of SWNT [30]), Titan yellow (allowing to complex heavy metal ions [31]) or Rhodamine B. As shown in the present study, such guest-like molecules also include doxorubicin, a known chemotherapeutic used to treat many cancer types [32,33,34]. Advantages of SRLS comprise high capacity for binding of guest-like molecules and a protective role as it prevents accidental drug release. The presented system can simultaneously bind many compounds which can fulfill different functions [31,35,36]. CR-type molecules can thus be considered as possible drug carriers. Since supramolecular systems can bind proteins [22,37], in particular antigen-complexed antibodies, these compounds could be used for targeted drug delivery. SRLS could also support drug excretion preventing its accumulation in tissues [38].

Combining the two above-described systems (SWNT and SRLS both having properties of a drug carrier), to create one system composed of SWNT dispersed with the use of SRLS would enhance drug-binding capacity and could extend the range of possible applications.

In the present study, doxorubicin (DOX), belonging to the anthracycline class, was used as a model drug. DOX is one of the most efficient drugs used in chemotherapy of different types of cancer [39].

Anthracyclines, which possess a planar tetracycline structure, intercalate between base pairs in DNA helix suppressing replication and transcription. However, DOX administration leads to depletion of endogenous antioxidants, thus increasing oxidative stress and promoting development of cardiomyopathies and cardiac insufficiency [40,41]. In spite of this, it is used for its high efficacy and wide spectrum of anticancer actions. Therefore, the search for efficient carriers of this drug, which would provide for its local action, is very important since they could improve efficacy of drug delivery and reduce toxic effects.

The DOX molecule, with planar structure and aromatic character, binds to the supramolecular ribbon formed by Congo Red molecules most likely via intercalation, similar to earlier studied Rhodamine B [33,34,36,42,43]. In a previous paper, the possibility of creating mixed supramolecular systems composed of CR and DOX was demonstrated with the use of dynamic light scattering (DLS). During DOX binding to CR, the fraction of free DOX molecules was observed to disappear due to binding (intercalation) to CR supramolecular ribbon [30].

DOX can also interact with the surface of carbon nanotubes. DOX binding to CNT surface involves a contribution of both π–π stacking and hydrophobic and electrostatic interactions [44,45]. Interestingly, some reports on the effect of the nanotube width on binding potential indicated that the larger the nanotube diameter (and the smaller the radius of curvature) the better the DOX binding onto its surface [20].

The presence of an amino group in DOX structure has a significant effect on physicochemical properties of this drug, including sensitivity to pH changes. The pKa value of the DOX amino group is 8.2 [20,42,46]. 

The aim of this study was to analyze possibilities of binding doxorubicin to a carrier system composed of single-walled carbon nanotubes dispersed with Congo Red. The research involved the analysis of DOX binding to CR, a model SRLS and the mechanism of CR and DOX binding to carbon nanotubes. Drug release from the carrier was also evaluated.

## 2. Results

### 2.1. Analysis of DOX Interaction with SWNT–CR

#### 2.1.1. DOX Binding to CR

Formation of stable CR–DOX complexes was visualized with the use of electrophoresis. In agarose gel (0.06 M veronal buffer, pH 8.6) DOX migrated towards cathode while both free CR and CR–DOX complexes moved towards anode. Five molar proportions of CR:DOX were analyzed of which 5:1; 2:1 and 1:1 proved optimal. When DOX was in excess (proportions CR:DOX 1:2 and 1:5), complexes were observed to precipitate and DOX excess migrated out of them.

Electrophoresis was also performed in 10% polyacrylamide gel without SDS in Tris/sodium barbital buffer (pH 7.0). Free CR and CR–DOX complexes migrated towards anode with different rates. This method was used to analyze three optimal molar proportions of CR:DOX which produced soluble complexes (CR:DOX = 5:1; 2:1 and 1:1).

**Figure 2:**
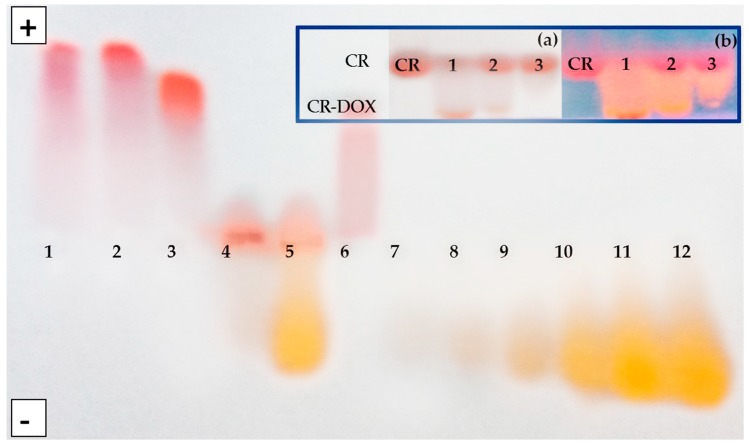
Agarose gel electrophoresis of CR–DOX complexes: (**1**) CR:DOX = 5:1; (**2**) CR:DOX = 2:1; (**3**) CR:DOX = 1:1; (**4**) CR:DOX = 1:2; (**5**) CR:DOX = 1:5, and (**6**) free CR and (7–11) free DOX at increasing concentrations corresponding to the concentration in complexes with CR. Insert: Electrophoresis in 10% polyacrylamide gel: (**1**) free CR and CR–DOX complexes: (**1**) CR:DOX = 5:1; (**2**) CR:DOX = 2:1; (**3**) CR:DOX = 1:1. It can be seen that CR–DOX complexes migrated faster than free CR. (**a**) visible light; (**b**) UV light (DOX better visible).

Migration rate of the obtained CR–DOX complexes was greater than that of free CR both in agarose and polyacrylamide gel. In polyacrylamide gel, separation is based both on the size and the charge. Positively charged DOX added to CR at 1:5 molar ratio produces the complex migrating faster than free CR. As DOX amount increased, complexes moved more slowly until their rate equaled the migration rate of free CR at the CR:DOX molar ratio 1:1. At CR:DOX molar proportion of 1:2 or higher, the charge was completely neutralized and complexes precipitated (Figure 2). 

Interestingly, binding a positively charged DOX by negatively charged CR produces CR–DOX complex that migrates faster towards the anode than free CR. It indicates that the complexes present properties which are not just an average of the two dyes. The explanation of this phenomenon (published in our previous paper and based on CR-Rhodamine B complexes) points to the fact that the conjugation of face-to-face stacked Congo Red molecules creates supramolecular ribbon-like structures which, in the electric field, become univocally oriented dipoles (due to the delocalization of pi electrons from stacked aromatic rings). Delocalization of electrons affects, in turn, the polar groups of Congo Red, changing their dissociation constants, and consequently the charge. This manifests itself as accelerated electrophoretic migration towards the anode upon increasing the concentration of CR, indicating to the concentration-dependent acidity of Congo Red [25]. In the case of DOX–CR complexes, the mechanism of the effect of complex formation on the charge is most likely the same. 

**Figure 3:**
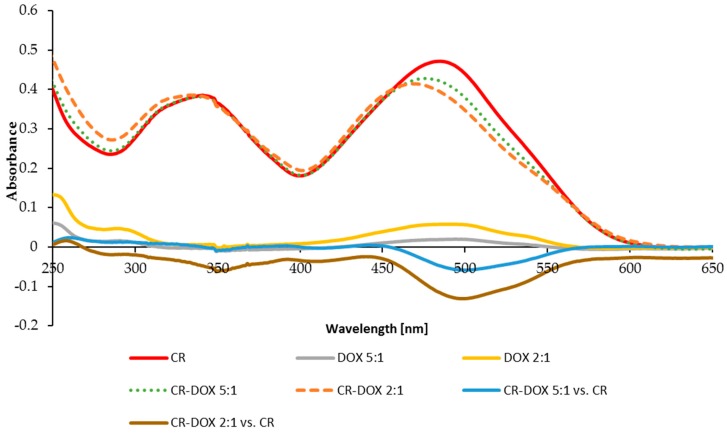
Hypochromic effect accompanying the formation of mixed supramolecular CR–DOX system. Spectra of DOX, CR and their complexes. CR:DOX molar ratios: 2:1 (dashed line) and 5:1 (dotted line) and free CR spectrum (red). DOX spectra: yellow—concentration corresponding to that in 2:1 complex and grey—concentration corresponding to that in 5:1 complex). Difference spectra for CR:DOX 5:1 measured against CR (blue) and for CR:DOX 2:1 complex measured against CR (brown).

Formation of CR–DOX complexes was accompanied by a change in absorption spectrum involving a hypochromic effect as compared to free CR spectrum, which indicated to a stronger interaction between aromatic rings of CR and DOX than between free CR molecules forming supramolecular ribbons (Figure 3). Two CR:DOX molar ratios were analyzed: 2:1 and 5:1. Spectra of free DOX at concentrations corresponding to its amount in complex 2:1 and 5:1 were also recorded and compared with corresponding difference spectra for CR–DOX 2:1 complexes measured against CR and for CR–DOX 5:1 complex measured against CR. 

The difference spectrum of DOX in CR–DOX complex measured against CR was compared with free DOX spectrum. A red-shift of the complexed DOX spectrum, measured in this way, was noted (λ_max_ change from 471 nm to 487 nm), which suggests participation of π electrons, i.e., binding of DOX by intercalation between CR molecules.

**Figure 4:**
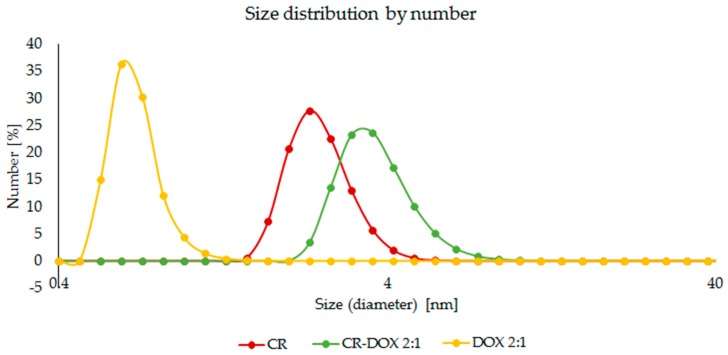
Dynamic light scattering analysis. Differences in the size of free DOX, free CR and CR–DOX complex. Size distribution by number. Concentrations of free CR and free DOX corresponding to that in CR–DOX complex.

The formation of CR–DOX complex was confirmed by dynamic light scattering (DLS) analyses. A distinct shift of CR–DOX peak (for the 2:1 sample) in relation to free CR and free DOX was observed indicating to the formation of aggregates of a larger size (Figure 4).

The above-described experiments proved the formation of a complex of self-assembled Congo Red molecules and molecules of doxorubicin. The interaction of both compounds was evidenced by a change in Congo Red absorption spectrum after DOX addition. The results of DLS analysis demonstrated that DOX was incorporated in the CR ribbon, increasing the size of the newly formed system. Electrophoresis showed that doxorubicin binding to CR changed the direction of its movement and Congo Red migration speed in electric field. Similar complexes with CR can also be formed by other compounds with flat molecular structure (binding of Rhodamine B, Evans blue, Titan yellow with Congo Red was described earlier) [35,36].

#### 2.1.2. DOX Binding to SWNT–CR

**Figure 5:**
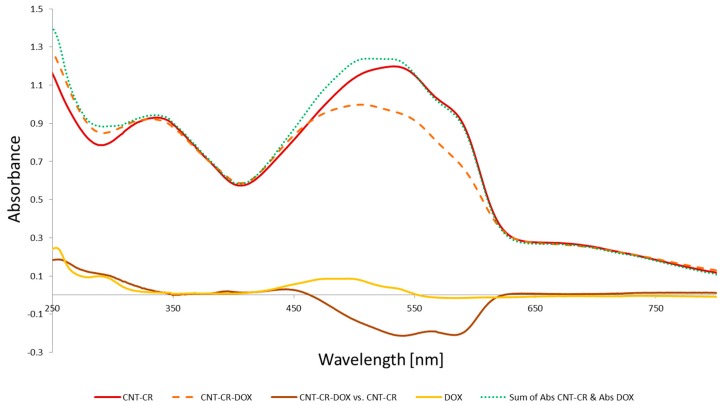
Spectra of DOX: DOX at concentration corresponding to that in 2:1 complex (yellow). Difference spectrum for SWNT:CR:DOX 2:1 complex (brown) measured against SWNT:CR complex (red).

The formation of SWNT–CR–DOX complexes was accompanied by a change in absorption spectrum involving a hypochromic effect as compared to SWNT–CR spectrum, which indicated to a stronger interaction between aromatic rings of CR bound on SWNT and DOX than between CR bound on SWNT (Figure 5). CR:DOX 2:1 molar ratios were analyzed. Spectra of free DOX at concentrations corresponding to its amount in complex 2:1 were also recorded and compared with corresponding difference spectra for SWNT–CR–DOX (CR:DOX molar ratio 2:1) complexes measured against SWNT–CR. The difference spectrum showed a red-shift of DOX absorption maximum (λ_max_ change from 471 nm to 526 nm), as in the difference spectrum of CR–DOX measured against CR. It indicates DOX binding to SWNT-CR complex and changes in electron flow in DOX molecule after binding to SWNT-CR [47].

**Figure 6:**
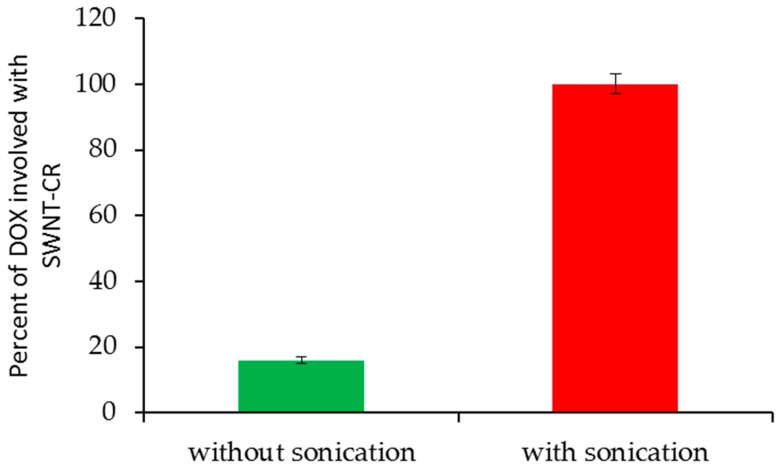
Influence of sonication on doxorubicin binding with SWNT-CR.

DOX binding into the Congo Red ribbon was proved by experiments that used ultrasounds to break supramolecular structures. The amount of DOX bound to SWNT–CR was evaluated (the total amount of DOX added to SWNT–CR was assumed to be 100%). After adding DOX to the free CR, the complex was created immediately. However, when DOX was added to the CR previously associated with SWNT, sonication of the SWNT–CR complex with added free DOX was required for complex formation (Figure 6). We can speculate that it can be explained by stabilization of CR ribbon-like structure after its binding to the nanotube surface leading to the decreased ability to bind DOX. Nanotubes used in this experiment were narrow enough (diameter 0.7–0.9 nm) not to bind DOX directly. Most probably, sonication temporarily weakens CR–CR interaction which facilitates DOX binding to CR molecules already bound to the nanotubes. Therefore, DOX binding occurred when Congo Red ribbon-like structure gradually reassembled after sonication. CR that is already bound to the nanotube forms a very compact structure (attached to SWNT surface) that prevents DOX intercalation which became possible when this structure was mechanically disturbed by sonication.

**Figure 7:**
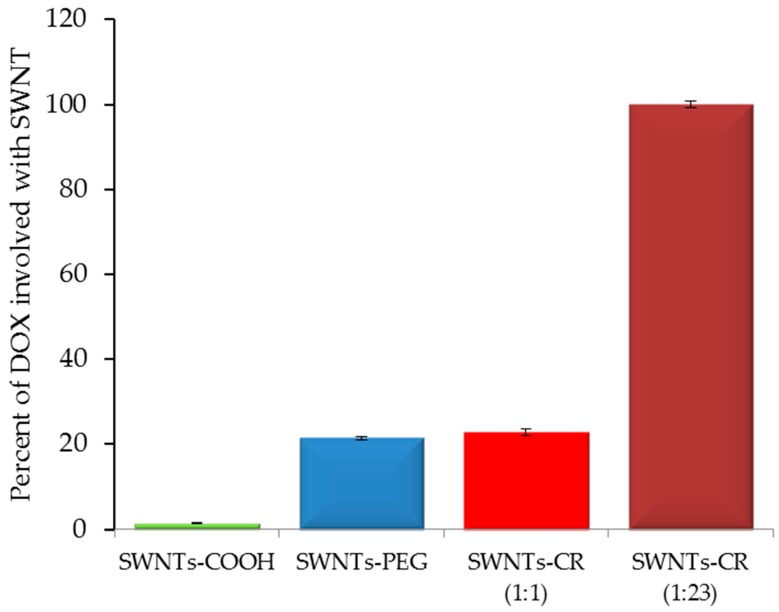
Interaction of DOX with differently functionalized SWNT comparison of the percentage of DOX added to the sample that formed a complex with nanotubes.

We compared the yield of DOX binding to nanotubes non-functionalized (SWNT) and functionalized in different ways: carboxylated (SWNT-COOH), PEG-coupled (SWNT-PEG) [20] and complexed with different amounts of CR: low (SWNT-CR weight ratio 1:1) and high (SWNT-CR weight ratio 1:23 proportion after removal of CR excess). Choosing only two weight ratios (1:1 and 1:23) was aimed to show differences between low and high levels of CR. CR at high concentrations presents different properties than at low concentrations. Increased concentration favors supramolecular association, resulting in longer ribbon-like structures with a pronounced dipole structure. This is evident in electrophoresis, where the rate of migration depends on the concentration of CR [25]. DOX binding to SWNT and SWNT–COOH was not observed while its binding to SWNT–CR (1:1) was at the same level as to SWNT–PEG. The result for SWNT–CR 1:23 was several times higher. Therefore, SWNT–CR complexes showed the highest efficacy of DOX binding especially those binding a large excess of CR. It means that functionalization with self-assembled ribbon-like compounds (SRLS), like Congo Red, allows for adjustment of the amount of DOX bound to the carrier, and to obtain, when needed, a high-capacity carrier system (Figure 7).

The sequence of steps in the SWNT–CR–DOX complex formation procedure is important. It was shown that the efficiency of DOX binding to SWNT was much higher if SWNT were first covered with CR, and DOX was added afterwards. Incorporation of DOX into CR before binding to SWNT decreased both CR-SWNT interaction and DOX binding.

Samples containing a constant amount of SWNT, CR and DOX (CR:DOX molar ratio of 18:1) were prepared. One series of the samples containing all three components added simultaneously was subjected to sonication while in the second series, the formation of SWNT–CR complex was formed before DOX was added. In order to assess DOX binding, all samples were filtered through AmiconUltra filters (permeable to DOX but impermeable to CR and SWNT), and then the concentration of the unbound DOX was measured spectrofluorimetrically in the obtained filtrate. Next, the samples with bound DOX, which did not pass through AmiconUltra filter, were filtered through PTFE membrane (permeable for CR and DOX but retaining SWNT) in order to evaluate whether they contained DOX bound with CR or with CR attached to SWNT. In addition, chromatographic analysis was performed for both the filtrates that have passed through the PTFE membrane and samples retained on these membranes (Table 1).

**Figure 8:**
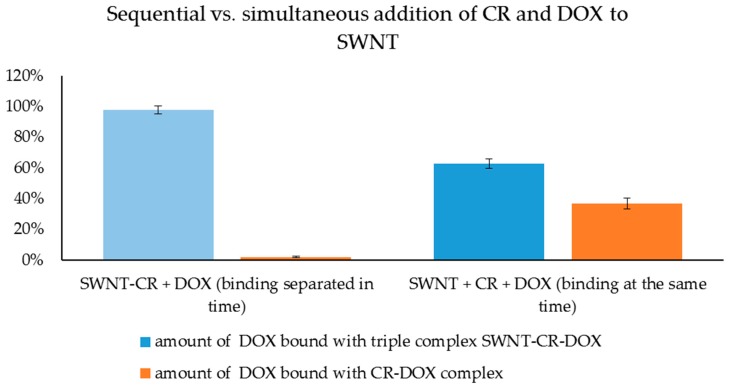
Sequential versus simultaneous addition of CR and DOX to SWNT. Percent of DOX added to the sample found in complexes.

It was shown that doxorubicin did bind to CR irrespective of the procedure used for complex formation because no free DOX was found in filtrates after filtration through AmiconUltra filters (results 1A,B in Table 1). To answer the question of whether DOX is bound to free CR or to CR attached to SWNT, the samples were filtered through PTFE membranes. Significant differences were observed. For samples in which binding of the components was separated in time, filtrates from PTFE filters contained neither CR nor DOX (result 2A, Table 1), while for samples in which all components mixed simultaneously (result 2B, Table 1), filtrates after PTFE filtration contained both CR and DOX (results 2B, Table 1). It indicates that separation of CR and DOX binding in time results in the formation of SWNT–CR–DOX complex and all doxorubicin was bound to SWNT–CR and did not occur in the form bound to free CR. It was confirmed by the results of chromatographic separation of the filtrate from PTFE filtration and of the material retained on the membrane (CR, DOX and SWNT were present) (result 3A, Table 1). In the second series, in which all three components were mixed simultaneously, PTFE filtrate contained both CR and DOX. Therefore, DOX could bind to both CR and CR-SWNT (result 2B, Table 1). This was confirmed by chromatographic analysis of PTFE filtrates and material on the PTFE membrane, where CR and DOX were found in both cases (result 3B, Table 1) (Figure 8). 

We can thus conclude that CR–DOX complex is strong enough and different from free CR, and it does not bind to the surface of carbon nanotubes.

Based on the results obtained in the above-described experiments, the possible mechanisms of CR and DOX binding with nanotubes were considered. In the studied system, free DOX did not bind to SWNT. It also did not bind spontaneously to the pre-assembled SWNT-CR complex. DOX could bind to SWNT-CR only after sonication, which led to the separation of all components. Most likely, DOX molecules intercalate into supramolecular ribbon-like CR structures forming on nanotube surfaces. However, it is important to note that when all three components (SWNT, CR and DOX) were sonicated simultaneously, DOX was not completely bound to SWNT–CR complex but it could also bind to free CR creating CR–DOX complex (present in PTFE filtrate). This can be explained by preferential intercalation of DOX into the ribbon formed by free CR, as the CR–DOX complex was less prone to binding to the surface of nanotubes. On the other hand, SWNT–CR complex formed without DOX, in which some CR molecules were tightly bound to the surface of nanotubes, and probably did not completely separate during the next exposure to ultrasounds, making DOX intercalation possible. It suggests that the intercalation occurs in supramolecular ribbon-like structure regions which are not directly bound to the surface of nanotubes but preferably in those that are more distant where intermolecular interactions are weakened by ultrasounds.

The mechanism of CR interaction with the SWNT surface was defined as face-to-face stacking due to observed changes in the CR spectrum after binding to SWNT [29]. The results of the experiment in which the SWNT–CR complex was bound to DOX, presented in this paper, provides the next argument for such interaction. We can speculate that during sonication of SWNT-CR complex, CR molecules directly bound to the nanotube surface were not liberated, but those bound indirectly (CR-CR) were released to the solution where they could create complexes with DOX. After sonication, CR–DOX complexes gradually bound to the CR already attached to nanotubes (face-to-face). However, if all components added simultaneously (free CR, free DOX and free SWNT) were sonicated, after sonication was completed, CR–DOX complexes were formed first which were strong enough to reduce their ability to interact with the SWNT surface compared with free CR.

**Figure 9:**
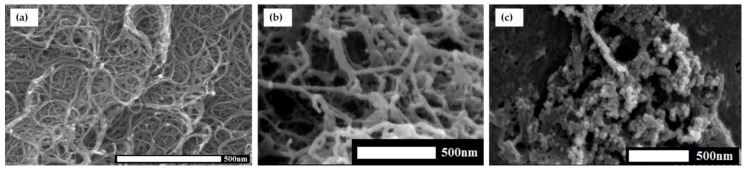
(**A**) SWNT; (**B**) SWNT-CR (1:5); (**C**) SWNT–CR–DOX—uneven loading of nanotubes by CR–DOX (SEM).

Single-walled carbon nanotubes interacted with CR and the created a complex with DOX. Scanning electron microscopy images confirmed the formation of non-homogenous associates around nanotubes showing differently loaded locations (Figure 9).

DOX binding to SWNT–CR complex led to shortening of nanotubes measuring ca. 400 nm in length and ca. 1.5–2 nm in width at a weakly CR–DOX loaded location. Shortening of nanotubes was probably a result of nanotube stiffening and breaking at locations of CR–DOX complex binding [30].

### 2.2. The Effect of pH on Stability of SWNT–CR–DOX Complex: A Possibility of DOX Release

Drug carrier efficacy is determined by its ability to release the content after reaching the target. Assuming that the presented system should deliver the drug directly to cancer tissue, the simplest mechanism of drug release from the carrier system appears to make use of the lowered pH occurring in cancer tissue. Lower pH is observed not only in extracellular spaces of cancer tissue but also in lysosomes and endosomes (pH 5.0). Hence, numerous studies on drug release from different carriers were carried out at low pH. Low pH is a characteristic feature of many cancer types (below the physiological value of 7.4 and dropping even to 5.7), which increases their metastasis and reduces anticancer response of the immune system [48,49]. Drug binding and release from the carrier surface due to pH changes seems to be effective, i.e., it protects healthy cells from undesired side effects of the drug which is bound to the carrier at the physiological pH, and is released at the reduced pH in cancer milieu or after endocytosis at the target site [46,50,51,52,53]. Stability of the presented carrier complex SWNT–CR–DOX and capability of drug release were analyzed with the use of the dialysis and differential scanning calorimetry. DOX release rates from SWNT–CR–DOX system at pH 5.0 and 7.4 were compared.

#### 2.2.1. DOX Release Evaluated by Dialysis

**Figure 10:**
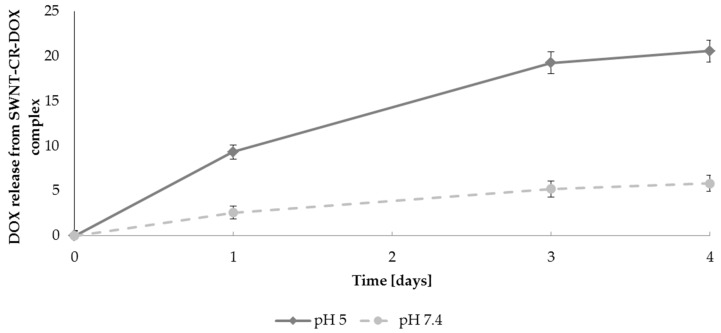
DOX release from SWNT–CR–DOX complex at pH 5.0 and at pH 7.4 at room temperature. The total amount of bound DOX was assumed to be 100%. Graphs present the mean values of three independent experiments. DOX concentration in dialysate measured spectrofluorimetrically.

DOX release from SWNT–CR–DOX complex depends on pH and was distinctly greater at pH 5.0 compared with pH 7.4 (Figure 10). This result indicates that lowered pH of endosomes or cancer environment can favor gradual DOX release from the carrier.

#### 2.2.2. DOX Release Evaluated by Differential Scanning Calorimetry (DSC)

The effect of pH on the ability to release DOX from the SWNT–CR complex was tested by differential scanning calorimetry (DSC). The studies of the SWNT–CR–DOX complex using the DSC method were carried out by recording changes in the heat flow rate during temperature changes in two heating cycles: heating after rapid cooling followed by heating after very slow cooling, allowing gradual ordering of the supramolecular structure. SWNT–CR–DOX samples (for concentrations respectively CR 2 mg/mL and DOX 2 mg/mL) were analyzed at pH 7.4 and pH 5.0. 

**Figure 11:**
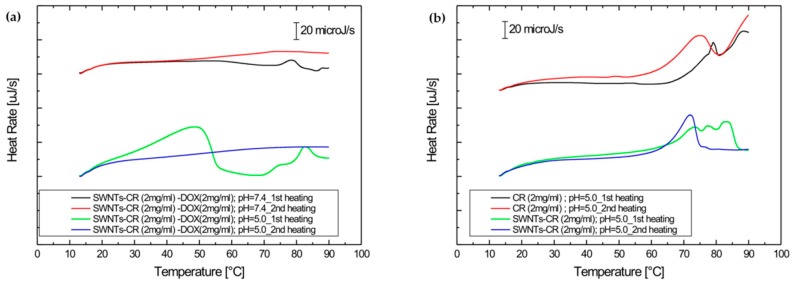
Calorimetric profiles: (**a**) SWNT–CR–DOX: two scans in the heating mode for each pH 7.4 and pH 5.0; (**b**) controls: CR and SWNT–CR—two scans for each in the heating mode at pH 5.0. The first heating was always preceded by uncontrolled rapid cooling, the second heating was always preceded by controlled slow (1 °C/min) cooling.

The results presented in Figure 11a indicate a high stability of the SWNT–CR–DOX complex at pH 7.4 during both heating cycles and its low stability at pH 5.0 during the first heating. This confirms the lowered stability of the complex at the reduced pH and DOX release. Lowering the pH causes the protonation of amine and sulfonic groups in CR molecules. Amino groups become positively charged, which affects the stability of the CR–DOX complex.

Controls for the above experiments were provided by thermograms obtained at pH 5.0 for free CR (2 mg/mL) and for SWNT-CR (CR concentration of 2 mg/mL) (Figure 11b). The results indicate that CR structure at reduced pH is very stable. The melting process was observed only in a high temperature range. The structure of SWNT–CR complex during heating at lowered pH also proved stable and its disintegration occurred at about 70 °C.

The phase transition observed 50 °C (Figure 11a) can be interpreted as DOX release from SWNT–CR–DOX complex at pH 5.0, as both free CR and SWNT-CR are stable at such conditions.

The results demonstrate that the system is stable at physiological pH but pH reduction results in slow DOX release, which can be advantageous from therapeutic perspective. These results are in agreement with similar earlier studies [46,48,52].

### 2.3. Evaluation of CR and SWNT-CR on Cell Proliferation

**Figure 12:**
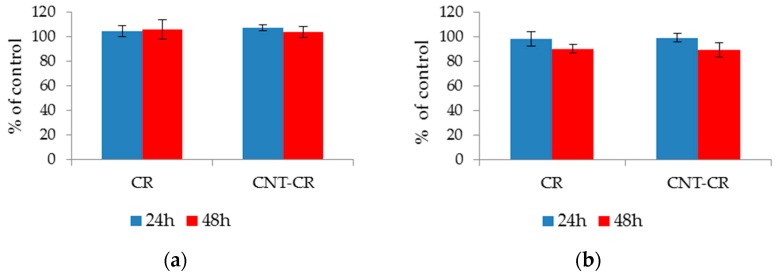
Proliferation of the cell lines (**A**) Hs27 and (**B**) U87MG treated with free CR and SWNT-CR for 24 and 48 h. Analyzes made using the crystal violet test. The results are expressed as a % of the control value (number of untreated cells after 24 or 48 h). The graphs show the average values of three independent experiments, each measurement point was made in three to five replications.

An ideal drug carrier should be characterized by low cytotoxicity and ensure normal cell proliferation. The effect of free CR and SWNT-CR complex on the proliferation of normal (Hs27) and tumor (U87MG) cells was checked. The results are presented as % of control (treated as the number of cells grown in the medium after 24 or 48 h). For both lines, the cells maintained proliferation at the control level (>90%), which indicates the biocompatibility of both the CR and the SWNT–CR complex (Figure 12).

## 3. Discussion

Targeted drug delivery to pathologically affected areas is especially important for drugs producing serious side effects. Drug carriers should complement properties of the drug or weaken its undesired effects, should show high capacity for the drug and enable targeted drug transport and controlled release [11,42,49].

In this paper, we present a mixed supramolecular system created by binding of the model drug DOX to Congo Red (CR) supramolecular ribbon-like structure (SRLS) which forms a complex with SWNT. The complex (carrier) created in this way, due to the presence of SRLS, is able to efficiently bind DOX and has an elevated binding capacity compared with nanotubes alone. SWNT constitute a scaffold-like structure for SRLS, which can reduce excretion rate of the drug–carrier complex from the body prolonging the time of drug action at the target site. Depending on the nanotube diameter, drugs can bind both to the nanotube surface directly and/or can interact with Congo Red molecules already bound to the nanotube surface [37,54]. It gives an opportunity to create in the future multipurpose high-capacity systems capable of binding simultaneously several compounds fulfilling different functions (e.g., drugs, metal ions, markers, etc.), which can significantly increase their attractiveness from the therapeutic perspective. However, the multipurpose high-capacity of the system remains to be proven [55]. In addition, mixed supramolecular systems preserve abilities of pure Congo Red to interact with proteins, which also can increase their potential usefulness as drug carriers [56,57].

The analyzed SWNT–CR complex has a wide range of potential applications. Advantages of carbon nanotubes (e.g., large binding area and scaffolding of SRLS-type compounds) were combined with benefits of Congo Red (ability to form supramolecular ribbon-like structure and to intercalate drugs or large binding area) to create a hybrid system endowed with properties of its both components. Advantages of the presented system include the ability to bind different compounds with planar aromatic structure (including drugs) and the possibility of controlled release at a reduced pH, typical of cancer tissues. Since the proposed hybrid system contains Congo Red, which can bind antibodies forming complexes with antigens or amyloids, and different compounds can intercalate into it, it creates an opportunity for administration of different drugs carried by SNWT–CR to inflammatory foci, wounds or amyloid plaques. It is also possible to modify the amount of bound supramolecular compound or to include another SRLS (Evans blue, Titan yellow) or even to use a combination of different compounds. 

Referring to previous developed in vivo delivery systems for doxorubicin using SWNT dispersed by PEG-lipids [20,58], several advantages of SWNT–CR–DOX system can be found. Thanks to the presence of self-assembling supramolecular Congo Red, which forms ribbon-like structures, we expect the effects of targeted drug delivery. CR binds to proteins and forms stable complexes. This interaction is possible in protein areas which were structurally destabilized—as in case of antigen-complexed antibodies [59]. The use of CR also significantly increases the binding capacity for doxorubicin, as carbon nanotubes form a scaffold, capable of binding a large amount of CR together with DOX [57].

Tests for the proliferation of normal and cancer cells treated with CR and its complex with SWNT performed in this work show the lack of in vitro toxicity of the systems used. Similarly, in vivo studies using CR to analyze interactions with antigen-antibody systems (rabbit ear tests) showed no CR toxicity and its complete removal from the body via the urinary tract [38,57].

The results show that the studied complex meets basic criteria indispensable for its potential application as a drug carrier able to reduce side effects of drugs and to increase their pharmacological efficacy.

## 4. Materials and Methods

### 4.1. Materials

Congo Red (CR, 96% purity, Aldrich Chemical Company, Inc. MILWAUKEE WI 53233 USA), single walled carbon nanotubes (SWNT, purity > 90%, length 700 nm, diameter 0.7–0.9 nm, SIGMA –ALDRICH, Co.,3050 Spruce Street, St. Louis, MO 63103 USA), doxorubicin hydrochloride (SIGMA –ALDRICH, Co.,3050 Spruce Street, St. Louis, MO 63103 USA) and sodium cholate hydrate (SIGMA –ALDRICH, Co.,3050 Spruce Street, St. Louis, MO 63103 USA). All other reagents used were of analytical grade and were purchased from commercial sources.

### 4.2. Preparation of CR–DOX and SWNT–CR–DOX Complexes

SWNT–CR complexes were obtained according to the procedure described previously [29] with the use of sonication and pressure-driven filtration through polytetrafluoroethylene (PTFE) membranes (0.2 μm pore size; MERCK Millipore). These membranes are impermeable to carbon nanotubes and do not bind CR but are permeable to the latter. The amount of CR bound to nanotubes was calculated from the known amount of CR added and by measuring absorbance of free CR in the filtrate at 489 nm (ε_489_ = 50.46 cm^−1^ mM^−1^). This procedure yielded well-dispersed carbon nanotubes complexed with Congo Red.

In order to bind DOX, doxorubicin hydrochloride was added to SWNT–CR complex produced in the above-described manner. The mixture was sonicated for 30 min in a cooled water bath. The sample was incubated for 24 h at room temperature and then filtered several times to remove excess of unbound DOX until a colorless filtrate was obtained. Filtration was performed in AmiconUltra filtration tubes (MWCO 50 kDa, MERCK Millipore Ltd., Tullagreen, Carrigtwohill Co. CORK IRELAND), which is impermeable to nanotubes and CR but permeable to DOX. The amount of DOX bound was calculated based on measuring fluorescence of free DOX in the filtrate (Ex = 470 nm, Em = 550 nm) and reading the result from a calibration curve. The results were confirmed by chromatographic analysis. Thin layer chromatography was run on silica gel (Silicagel 60, Merck, Darmstadt, Germany) in butanol: water: methanol (5:2:3) solvent, which allowed for separation of SWNT, CR and DOX. The amount of DOX was measured spectrofluorimetrically after elution from the gel. 

### 4.3. Characterization of CR–DOX or SWNT–CR–DOX Complexes

#### 4.3.1. Electrophoresis

Electrophoresis was performed in 1% agarose gel (0.06 M sodium barbital buffer pH 8.6) or in 10% polyacrylamide gel without SDS in Tris/sodium barbital buffer (pH 7.0).

#### 4.3.2. Dynamic Light Scattering (DLS)

Dynamic light scattering (DLS) detector (DynaPro MS800 Protein Solutions, Ltd. High Wycombe, England) was applied in the measurements of hydrodynamic radius of CR and DOX. The ability to form a CR–DOX complex was also evaluated. The 25 °C measurement temperature was applied after a 3 min incubation inside the instrument. The measurement of each sample was done in three replicates after 30 acquisitions lasting 10 s each.

#### 4.3.3. Scanning Electron Microscopy (SEM)

Scanning electron microscopy (SEM; Hitachi S-4700) was used to evaluate the changes in the structure of the carbon nanotubes caused by the binding of CR and CR–DOX complexes. Free carbon nanotubes associated with CR or associated with the CR–DOX complex were applied to a culture dish. Then a 2% glutaraldehyde in PBS solution was added to fix the samples. Then it was rinsed with phosphate buffer, stabilized in 2% osmium tetraoxide in phosphate buffer, rinsed and dehydrated in increasing concentrations of ethyl alcohol. The samples were dried and sprayed with technical gold in a vacuum sprayer. The samples thus prepared were analyzed in an electron scanning microscope.

#### 4.3.4. Effect of pH on DOX Release Dialysis 

The kinetics of DOX release from SWNT–CR–DOX complex at different pH was determined by dialysis using D-Tube^TM^ Dialyzers Mini, MWCO 12–14 kDa (Novagen, MERCK Millipore, EDM Millipore Corp., Billerica, MA USA). SWNT–CR–DOX samples were dialyzed to acetate buffer (0.05 M, pH 5.0) or to Tris-HCl buffer (0.05 M, pH 7.4). At several time points, samples of dialysis fluid were collected for analysis, and were replaced with the same portions of the appropriate fresh buffer. The amount of released DOX was measured spectrofluorimetrically using a calibration curve. 

#### 4.3.5. Differential Scanning Calorimetry (DSC)

Differential scanning calorimetry (DSC) was used to evaluate stability of the system and to assess the effect of pH on DOX release from SWNT–CR complex. Studies of SWNT–CR–DOX complex using DSC was carried out by recording changes in heat flow rate during temperature changes in two heating cycles, i.e., heating after rapid cooling and then heating after very slow cooling, allowing for gradual formation of supramolecular structure. SWNT–CR–DOX samples (for concentrations of CR 2 mg/mL and DOX 2 mg/mL) were analyzed at pH 7.4 (0,05 M Tris/HCl buffer with addition of 0,264 M NaCl) and pH 5.0 (0.05 M acetate buffer with addition of 0.264 M NaCl). The tests were performed using a NANO DSC Series III System (Model 6300) with Platinum Capillary Cell (TA Instruments, 159 Lukens Drive, New Castle, DE 19720, USA) equipped with Nano DSCRun software. To avoid the formation of air bubbles during heating, the samples were degassed for 10–15 min before measurement, using a vacuum of 0.3–0.5 atm. The sample was then placed in a capillary measuring cell with a volume of 0.33 mL, while the reference chamber was filled with the buffer in which the analyzed sample was dissolved. All measurements were made at 3 bar. Data were recorded in the range of 10–90 °C, with a scanning speed of 1 °C min ^−1^, both during heating and cooling. To ensure a thermodynamic equilibrium, the measurement system was equilibrated for 10 min. at the initial temperature (10 °C in heating mode and 90 °C in cooling mode). The baselines were made by recording scans for reference samples (appropriate buffers) that were simultaneously filled in the sample and reference chamber. Each data set was analyzed using the NanoAnalyze software provided by TA Instruments.

### 4.4. Evaluation of Cell Proliferation after Addition of CR and SWNT–CR

Cells of the U87MG and Hs27 lines were plated into 96 well plates at 10,000 cells/well. The cells were grown in DMEM medium with 10% FBS for 24 h to adhere to the plate surface, then rinsed with phosphate buffer (PBS) and fresh medium added. CR (80 μM) or SWNT-CR (concentration of CR: 80 μM) were added to the culture. The culture was then carried out for 24 or 48 h. Proliferation was assessed using a colorimetric crystal violet assay. The method of the colorimetric crystal violet test (hexamethyl-p-rosaniline hydrochloride) is based on the staining of live cell nuclei growing on the surface of the culture vessel after rinsing the dead cells.

After incubation, the medium was removed, cells were washed with phosphate buffer—PBS (without Ca ^2+^/Mg ^2+^, 0.05 M, pH 7.4) and then fixed for 15 min with methanol. After fixing the cells, removing methanol and drying the plates, the cells were stained with a 0.5% solution of crystal violet (Sigma Aldrich) in a 20% aqueous methanol solution for 3 min. The excess dye was then removed, the plates washed with distilled water and the decolorizer solution (sodium citrate, citric acid in a 50% methanol solution) added over 20 min. The absorbance of the obtained solutions measured at 540 nm was a measure of the number of cells. This number was presented as % of control (untreated cells cultured in DMEM medium with the addition of 10% FBS, for a given time, 24 or 48 h).

## Figures and Tables

**Table 1:** The effect of sequence of steps in the SWNT–CR–DOX complex formation procedure.

Binding of SWNT, CR and DOX Separated in time		Simultaneous Binding of SWNT, CR and DOX	
**SWNT + CR** **Sonication 1** **+ DOX** **Sonication 2**		**SWNT + CR + DOX** **Sonication**	
**Filtration (AmiconUltra);** *present in filtrate:*			
*CR*	*-*	*CR*	*-*
*DOX*	*-*	*DOX*	*-*
**RESULT (1A):** All DOX bound to SWNT-CR or to free CR		**RESULT (1B):** All DOX bound to SWNT-CR or to free CR	
**Filtration (PTFE);** *present in filtrate:*			
*CR*	*-*	*CR*	*+*
*DOX*	*-*	*DOX*	*+*
**RESULT (2A):** Lack of DOX and CR in filtrate All DOX and CR bound with SWNT		**RESULT (2B):** CR and DOX present in filtrate DOX bound with free CR and/or with SWNT-CR	
**Chromatography (after filtration through PTFE)**			
*Chromatographic analysis of filtrate:*			
*CR*	*-*	*CR*	*+*
*DOX*	*-*	*DOX*	*+*
*Chromatographic analysis of complexes bound to SWNT, retained on the membrane:*			
*CR*	*+*	*CR*	*+*
*DOX*	*+*	*DOX*	*+*
**RESULT (3A):** SWNT–CR–DOX complexes present on PTFE membrane DOX was bound by SWNT-CR		**RESULT (3B):** DOX bound with free CR but also with SWNT-CR complexes	

## Abbreviations

SRLS	Self-assembled ribbon-like structure
CR	Congo Red
CNT	Carbon nanotubes
SWNT	Single wall carbon nanotubes
DOX	Doxorubicin
MDR	Multidrug resistance
DLS	Dynamic light scattering
PTFE	Polytetrafluoroethylene
SEM	Scanning electron microscopy
DSC	Differential scanning calorimetry
